# Vapor Trace Recognition Using a Single Nonspecific Chemiresistor

**DOI:** 10.3390/s130709016

**Published:** 2013-07-12

**Authors:** Vladimir Dobrokhotov, Alexander Larin, Dewayne Sowell

**Affiliations:** Department of Physics and Astronomy, Western Kentucky University, Bowling Green, KY 42101, USA; E-Mails: alexander.larin375@topper.wku.edu (A.L.); dewayne.sowell363@topper.wku.edu (D.S.)

**Keywords:** sensor, electronic nose, pattern recognition, transient response, LDA, QDA, 73.63.Bd, 73.63.Rt

## Abstract

An application of spectral analysis to the transient response signals of ALD-fabricated conductometric sensors (chemiresistors) upon exposure to short vapor pulses is discussed. It is based on the representation of a response curve in the frequency domain, followed by the multi-dimensional Quadratic Discriminant Analysis (QDA) for analyte identification. Compared to the standard steady-state amplitude analysis, this technique does not depend on a short-term sensor drift, does not have limitations for the number of extracted features and has a strict physical validation. Effective recognition of some relatively simple combustible analytes (acetone, toluene, ethanol) was demonstrated using a single nonspecific chemiresistor.

## 1. Introduction

Detection and recognition of combustible and explosive vapors remains an important problem for industry and national security. Major efforts in this direction have been concentrated on the development of analytical instruments, such as spectrometers of different kinds that are capable of detecting and analyzing the molecular structure of gaseous species. Currently, analytical tools of this type remain bulky and expensive, require considerable time for analysis, typically under laboratory conditions, and highly-qualified personnel to operate them. The best alternative for replacing the spectroscopy tools is the use of artificial olfactory systems also known as electronic noses [1,2]. An electronic nose is a biologically inspired device that identifies and analyses chemical compounds in gaseous environments. An electronic nose consists of a mechanism for chemical detection, such as an array of electronic sensors, and a mechanism for pattern recognition, such as a neural network [3].

**Figure 1.**
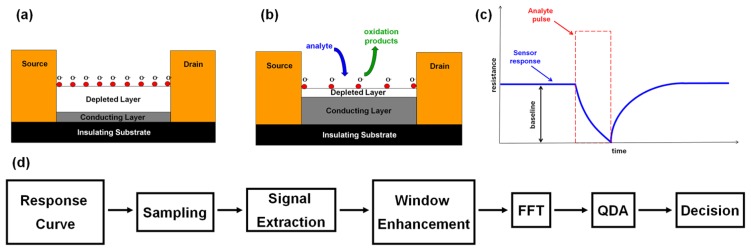
**(a)** Schematics of a thermally activated metal oxide chemiresistor of with O^−^ termination at the surface in the ambient air and (**b**) upon the exposure to combustible vapor. (**c**) A typical response curve of a metal oxide chemiresistor upon exposure to a combustible vapor (**d**) Stages of signal processing and pattern recognition.

Conductometric sensors (chemiresistors) are traditionally used as the building blocks for electronic noses [1,2]. A chemiresistor is a device whose electrical resistance can be modulated by molecular adsorption on its surface. Typically, the changes in resistance are proportional to the partial vapor pressure of chemicals in the atmosphere; hence a chemiresistor converts the concentration of chemicals in the atmosphere into a measurable electrical signal. Commonly utilized chemically sensitive materials are carbon-black polymer composites, arrays of metal nanoparticles, biological materials and metal oxide films [4–16]. Sensing behavior is one of the most important and well-known properties of metal oxide materials and it was found that metal oxide sensors usually demonstrate much higher sensitivity, selectivity, and stability to their chemical environment than the other materials. The sensing mechanism by metal oxide films is primarily based on high-temperature activation of atmospheric oxygen on the semiconductor surface [4–13]. Consequently, the catalytic reactions of gaseous species with oxygen sites on the surface induce charge transfer from the surface to the bulk, changing the electrical resistance of the device (Figure 1a–c)). In order to generate a comprehensive smell-print, sensors are usually arranged in an integrated array. Individual chemiresistors in the array are nonspecific and vary by physical or chemical parameters, such as: type of oxide, thickness of oxide layer, temperature, *n* or *p*-type volume doping, surface doping with catalytic nanoparticles.

Upon the exposure to analyte, the integrated patterns of signals undergo processing by the pattern recognition software. Most feature extraction techniques for recognition software have been based on linear techniques, mainly principal components analysis (PCA) and Fisher's linear discriminant analysis (LDA) [3,17]. PCA is a signal representation technique that generates projections along the directions of maximum variance, which are defined by the eigenvectors of covariance matrix. LDA is a signal classification technique that directly maximizes class separability, generating projections where the examples of each class form compact clusters and the different clusters are far from each other. The LDA algorithm selects features that are most effective for class separability, while PCA selects features important for class representation. Initially, the most common practice for recognition of gaseous analytes was to use the steady state responses of the sensors as a feature vector [1–3,17]. At the same time, the transient response contains sufficient discriminatory information. Later, multiple successful studies have been conducted in order to expand the LDA and PCA for transient response. In these studies, the feature vector was extracted using a variety of methods, such as fast Fourier transform (FFT) [17], Continuous wavelet transform [18], Gardner transform, multi-exponential transient spectroscopy, Pade-Laplace and Pade-Z transforms [19], and dynamic moments [20]. In an earlier work [21] the authors demonstrated the extraction of a transient feature, which is strongly correlated with the steady-state response, but available much earlier than the latter. Once the features are extracted, the back-propagating PCA or LDA-based neural network is typically used for predictive classification.

There is no universal sensor system that can solve all the gas or vapor analysis problems. Instead, there is a need to employ intelligent sensor systems that are appropriate to the application. This means building in intelligence through the development of suitable sensor structures, sensor materials and pattern recognition methods. In this paper we present a technique that allows one to build a simple vapor recognition system based on a single broadly-tuned metal-oxide chemiresistor, fabricated using atomic layer deposition (ALD). This method is based on the combination of fast Fourier transform (FFT) with exponential windowing and Quadratic Discrininant Analysis (QDA). It is specifically designed for rapid delivery of the analyte to the sensor in the form of short 0.1 s bursts and takes advantage of the rising and declining parts of the response curve. In contrast to the traditional analysis of the steady-state responses, the advantage of this method is the ability to analyze the entire catalytic process occurring on the sensor. Sensor calibration was performed *via* a VaporJet calibrator, which simulates vapor-pulse delivery typical for pre-concentrators of analytes [1,22]. This approach has strict physical validation, since the time-dependent features of the response curve are uniquely determined by a particular catalytic reaction. The number of extracted features in our method does not have limitations and can be adjusted depending on tested analytes.

## 2. Experimenal Design

Chemiresistors were fabricated using a Beneq atomic layer deposition (ALD) system TFS 500 for deposition of ZnO on the silicon oxide substrates. ALD utilizes a binary reaction sequence of self-saturating chemical reactions between gaseous precursor molecules and a solid surface to deposit films in a monolayer-by-monolayer fashion. ALD of ZnO was conducted at 150 °C using diethyl zinc (DEZ) and deionized water (H_2_O) as zinc and oxygen sources, respectively [23–26]. During the deposition, the background pressure of 1 Torr in the reaction chamber was maintained by a steady flow (300 sccm) of the process gas nitrogen. Each ALD reaction cycle included a 250 ms DEZ pulse, followed by a 2 s pressurizing with nitrogen purge, a 250 ms H_2_O pulse and another 2 s nitrogen purge. A complete layer deposition consisted of 375 cycles. Following the above procedure, a uniformly distributed 85 nm polycrystalline ZnO coating was achieved.

**Figure 2.**
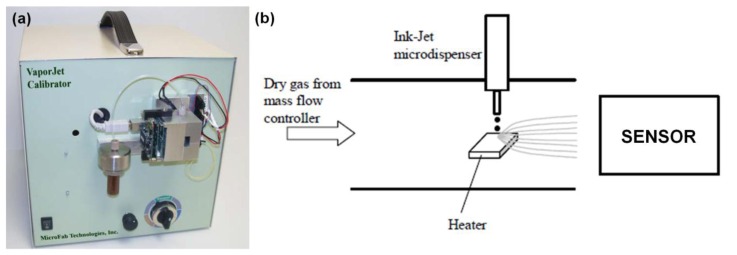
VaporJet calibrator (**a**) and principle of operation (**b**).

A standard two electrode test geometry was used for measuring the electrical response of chemiresistor to chemical vapors. Electrodes were deposited on the ZnO layer using shadow masking and consisted of the adhesion 50 nm thick nickel layer followed by 250 nm layer of gold. The electrodes were annealed and the ohmic nature of ZnO-metal contacts was verified by I–V characterization. The 5 mm × 5 mm sensor was connected to a thermocouple and placed on a variable temperature platform for temperature control. Sensor responses were acquired with a Keithley 3706 six-slot system switch with a high-speed multiplexer Keithley 3723 card interfaced to a computer via Labview-operated data acquisition software for real time conductance measurements. The sensor's operational temperature range was found to be between 100 °C and 500 °C. The lack of sensitivity below 100 °C is due to a sufficient drop in surface oxygen vacancies below this temperature. The reduction of sensitivity above 500 °C is caused by the activation of surface phonons resulting in a sufficient increase in the oxygen desorption rate. Maximum sensor response was achieved at 400 °C. The sensor was initially heated to 400 °C in synthetic air at ambient pressure to obtain a steady state resistance. Once the steady state resistance was achieved, pulses of vapors were introduced in the air flow by a MicroFab VaporJet calibrator (Figure 2a).

The principle of operation of the VaporJet calibrator is shown in Figure 2b. The ink-jet dispenser that is employed in the VaporJet calibrator is drop-on-demand (DOD), meaning that it can produce a drop only when required. The actuation of the dispenser is done with a piezoelectric element. The dispenser consists of a glass tube having an orifice at one end and being connected by the means of Teflon™ tubing to the reservoir containing the solution to be dispensed. An annular piezoelectric element poled in the radial direction is bonded to the glass using a thin epoxy layer. When voltage is applied to the piezoelectric actuator, the actuator contracts or expands (depending on the direction of the electric field produced in the piezoelectric element relative to the poling direction) and this deformation is transmitted to the glass and then to the fluid. Because the structural response is very fast and the solution to be dispensed is in contact with the glass, the motion of the structure translates in a volumetric change in the solution. The change produces a localized pressure variation that travels as acoustic waves in the solution contained by the glass tube. The details of VaporJet design and operation can be found in [22]. Thanks to the advantages of this technology, short 0.1 s pulses of various analytes were used for sensor exposure in this study.

## 3. Signal Processing and Pattern Recognition

**Figure 3.**
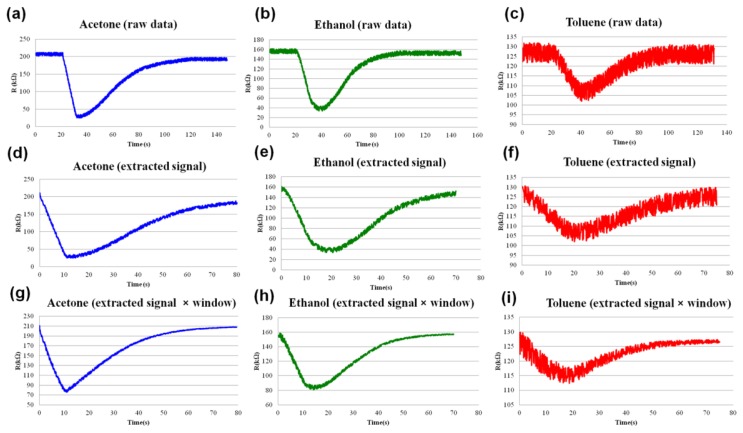
**(a–c)** Raw data obtained from the sensor. (**d–f**) Signal extracted by the analysis of autocorrelation coefficient (**g–i**) Extracted signal enhanced with the exponential window.

**Figure 4.**
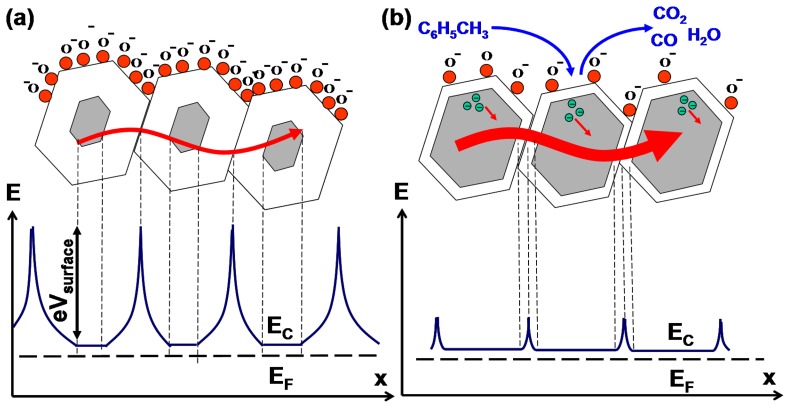
Schematic energy-level diagrams of the polycrystalline ZnO layer (**a**) prior to exposure to toluene and (**b**) after exposure and subsequent oxidation of toluene.

The signal processing and pattern recognition techniques are demonstrated on the example of three combustible substances: ethanol, toluene and acetone. All three of the tested chemicals effectively participate in a rapid catalytic oxidation on the surfaces of metal oxides. In the present study, the partial vapor pressures of the analytes in the ambient atmosphere were between 100 and 150 ppm. The raw data curves obtained from the sensors upon the exposure to 100 ppm of toluene, acetone and ethanol are shown in Figure 3a–c. Typically, the relative change of the sensor resistance in the range 0.1–10 times can only be achieved after a few seconds of exposure [10–16]. A significant change in resistance of the atomically deposited ZnO layer upon the 0.1 s exposure of 100 ppm analyte pulse can be broadly categorized as a finite size effect. Specifically, the diffusion of the molecular species between the neighboring nanocrystals, as opposed to depletion of free carriers over the entire surface of an individual ZnO nanocrystal, creates stochastic contact potentials at grain boundaries (Figure 4). The contact potential can be viewed as a tunneling barrier to electrons, which in our case is modulated by the oxidation of the analyte. It is well known that the surface area and the surface defects are crucial for a high sensitivity given that the ionization happens primarily on the edges and corners of the nanocrystals [4–6]. This explains the superior sensitivity of the ALD fabricated polycrystalline oxide films. The amplitudes of sensor responses are clearly different for different chemicals, which gives a discrimination mechanism for pure analytes having the same vapor pressure; however, the amplitude data can be insufficient if the vapor pressures are different. In addition, a common sensor drift problem makes vapor recognition by amplitude using a single nonspecific chemiresistor impossible.

**Figure 5.**
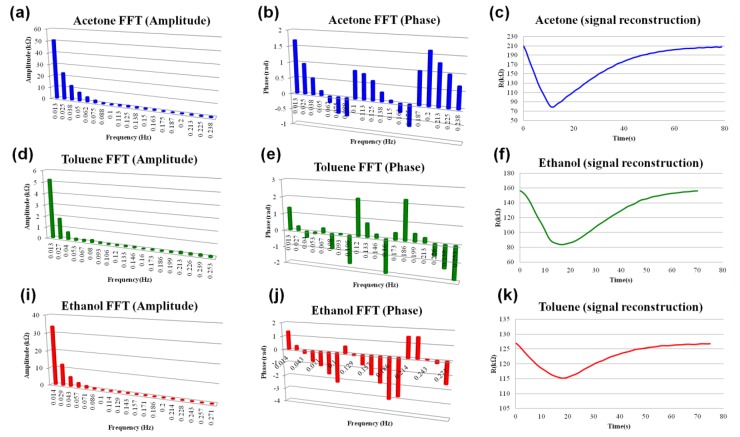
Spectral structure of the first twenty harmonics and the signal reconstruction in time domain for acetone (**a–c**), ethanol (**d–f**), and toluene (**i–k**).

**Figure 6.**
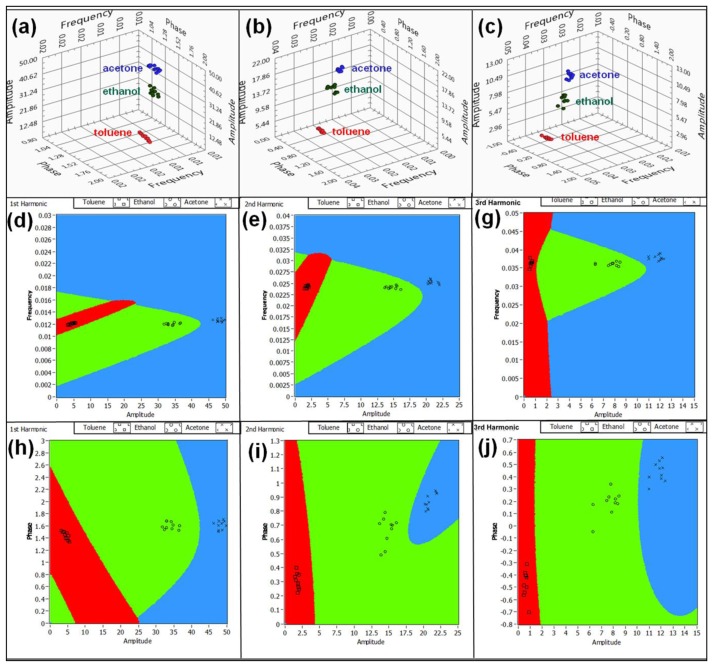
Visualization of the projections of 60-dimentional hyperspace (a–c). Clouds of points corresponding to different analyte exposures in 3-D (amplitude, frequency, phase) coordinate systems, corresponding to the first (**a**), second (**b**) and third (**c**) harmonics. Visualization of the quadratic discriminant analysis for the first (**d**,**h**), second (**e**,**i**) and third (**g**,**j**) harmonics.

A more productive approach would be to represent the entire response signal as a mathematical object suitable for processing by the recognition algorithm. The stages of signal processing and pattern recognition are shown in Figure 1d. In order to analyze the sensor response, the meaningful part of the response curve corresponding to the catalytic reaction on the surface has to be extracted. The extraction of meaningful part can be divided in two stages. A drop of resistance, corresponding to vapor exposure, is a relatively fast process compared to the rising part of the curve, corresponding to the recovery of oxygen species on the chemiresistor surface (Figure 1c). By monitoring the time derivative of the curve, the moment of rapid resistance drop can be detected. This moment is a starting point of the extracted signal. The sensor recovery takes longer, compared to the reaction phase. Therefore, the ending point of the extracted signal was determined by the analysis of an autocorrelation coefficient [27]:

(1) $$r_{a}=\frac{\sum_{i=1}^{N-k}\left(x_{i}-\bar{x})(x_{i+k}-\bar{x}\right)}{\sum_{i=1}^{N}{\left(x_{i}-\bar{x}\right)}^{2}}$$

where *x_i_* is a single observation, and $\bar{x}=\sum_{i=1}^{N}x_{i}$ is the overall mean. In our experiments a short-term autocorrelation was considered (*k* = 1) and the mean value was taken over *N* = 20 observations. The value of the autocorrelation coefficient that separates the signal from background noise depends on variety of factors corresponding to the particular experiment: type of background, humidity, temperature, or presence of electromagnetic field. Under the laboratory conditions the optimum lower boundary for autocorrelation coefficient that separates signal from noise was found to be 0.9. The meaningful part of the curve corresponding to sensor response to vapor exposure (extracted signal) is shown in Figure 3d–f. In order to create an array of input parameters for recognition algorithm, these raw data curves have to be transformed into mathematical objects suitable for processing. A very efficient technique to mathematically reconstruct the signal is to use fast Fourier transform (FFT). Application of FFT requires that the signal must be periodic in the sample window or frequency leakage will occur [27]. In other words, the signal must start and end at the same point in its cycle. Since the starting and ending points of them have different magnitudes (Figure 3d–f), FFT cannot be applied to the extracted part of the response curve directly. To preserve the spectral structure of the signal, the exponential window typical for response signals was applied. The exponential windowing is a multiplication of a response signal by the following function:

(2) $$w(n)=e^{-\left|n-\frac{N-1}{2}\right|\frac{1}{\tau }}$$

where *N* represents the window width as a number of counts, n is an integer with values 0 ≤ n ≤ N–1, and *τ* is the time constant of the function. The effect of the exponential window on the response signal in the time domain is shown in Figure 3g,h,i. The window weights the beginning and end of the sample to baseline so that it is more periodic during the FFT process. Upon applying the FFT to the enhanced data set, the response signal in a frequency domain is shown in the Figure 5. The obtained harmonics can completely reconstruct the signal in time domain as Fourier series. The example of signal reconstruction for acetone, ethanol and toluene using the first twenty harmonics is shown in Figures 5c,f,k. The contribution of baseline to the sensor response was eliminated as constant, which makes the extracted features independent from the short-term sensor drift. Evolution of features under long-term sensor drift [28] has not been evaluated in the frame of this study. Reconstructed response signals in the time domain (Figure 5c,f,k) are mathematical functions of the following type:

(3) $$x(t)=A_{0}+\sum_{n=1}^{N}A_{n}\cos(n\omega_{n}t+\theta_{n})$$

where *A_0_* is a baseline dependent constant and *N* is a number of harmonics. *A_n_*, *ω_n_*, *θ_n_* are spectral amplitudes, frequencies and phases respectively. For *N* = 20 the response signal can be characterized by 60 parameters: 20 amplitudes, 20 frequencies and 20 phases. Hence, in 60-dimentional hyperspace the entire signal can be represented as a single point. Introduction of pulses of different concentrations (between 100 and to 150 ppm) during the training process causes slight deviations in the spectral structure of the signal, which causes a formation of a cloud, rather than a single point. Upon the completion of training, the electronic smell-print of each chemical is represented as a cloud of points in 60-dimensional hyperspace. Figure 6a,b,c illustrate the 3-D subspaces of the first three harmonics out of twenty. As one can see, the data related to different analytes are clearly grouped. Based on the training of our device, the pattern recognition algorithm was developed. A standard approach in recognition of chemical patterns is a linear discriminant analysis (LDA) [1,3,5,6,17]. However, the major difficulty of LDA is the assumption that the Gaussian densities for different classes (analytes) have the same shape, but are shifted versions of each other (different mean vectors). Frequently, for chemiresistors this assumption is not supported by the experimental evidence and the linear boundaries between the classes are not always consistent. An improved version of LDA is the quadratic discriminant analysis (QDA), where the differences in distributions are included in the model and the classes are separated by the second order surfaces. The QDA algorithm applied to the vapor recognition problem can be introduced using the following formalism. Let *X⃗* be an arbitrary response signal vector of 60-dimensional hyperspace. Let *f_k_*(*X⃗*) be a probability that vector *X⃗* belongs to class *k*, and *f_l_*(*X⃗*) be the probability that vector *X⃗* belongs to class *l*. Assuming that the priory probabilities of *X⃗* ∈ *k* and *X⃗* ∈ *l* are equal, the boundary separating classes *k* and *l* is a 60-dimentional surface on which the probabilities *f_k_*(*X⃗*) and *f_l_*(*X⃗*) are equal to each other. Therefore, the equations for boundary between the classes *k* and *l* can be written in the following form [3]:

(4) $$\{\begin{array}{c} f_{k}(\vec{X})=\frac{1}{{\left(2\pi \right)}^{p/2}{\left|\Sigma_{k}\right|}^{1/2}}e^{-\frac{1}{2}{\left(\vec{X}-{\vec{\mu }}_{k}\right)}^{T}\sum_{k}^{-1}\left(\vec{X}-{\vec{\mu }}_{k}\right)}\\ f_{l}(\vec{X})=\frac{1}{{\left(2\pi \right)}^{p/2}{\left|\Sigma_{1}\right|}^{1/2}}e^{-\frac{1}{2}{\left(\vec{X}-{\vec{\mu }}_{l}\right)}^{T}\sum_{l}^{-1}\left(\vec{X}-{\vec{\mu }}_{l}\right)}\\ \frac{f_{k}(\vec{X})}{f_{l}(\vec{X})}=1 \end{array}$$

where *p* is the dimension, Σ*_k_* and Σ*_l_* are the covariance matrices, and *μ_k_* and *μ_l_* are the mean vectors of classes *k* and *l* respectively. The visualization of the QDA in 3-D subspace of the first 3 harmonics for acetone, toluene and ethanol are shown in the Figure 6d–j. The lines in Figure 6d–j separating the analytes are the projections of a 60-dimensional surface on coordinate planes of 3-D subspace. For QDA the projections are the second order curves, compared to LDA where the projections are linear. Once the training is complete and the boundaries are found, any new sensor response vector *Y⃗* will be attributed to a particular class of analytes.

The number of classes, as well as the structure of boundaries between the classes, is completely determined by the training data set. Therefore, with an increase in the number of training inputs, the structure of boundaries will evolve and eventually stabilize. For very different chemicals like acetone, ethanol and toluene at 100 ppm levels of concentrations the electronic smell-prints are perfectly separable. However, for similar analytes, like DNT and TNT, at lower levels of concentrations (ppb, ppt), the smell-prints will be less distinguishable and the statistical analysis of an integrated signal from multiple sensors with different catalytic properties will become essential. The extension of this method for multi-sensor detection will be considered in our future publications.

## 4. Effect of Humidity on Sensor Performance

One of the most complex problems of current sensor technology is the common issue of sensor drift due to the environmental conditions. Because of this, steady-state amplitude analysis has the potential to lead to false positives if the recognition algorithm training was conducted at a different humidity level than field conditions. This effect is especially well pronounced at low analyte concentrations, where drift becomes comparable with the sensor response. In contrast, the spectral analysis of the transient response is significantly more robust. In order to illustrate this point, vapor recognition using a transient response of a single chemiresistor was compared with recognition using the steady-state response of an integrated system of three chemiresistors with different thicknesses of sensing metal oxide layers. The discrimination between the acetone and ethanol at 10 ppm concentration levels was studied at different humidity levels. Water vapor was introduced in the ambient air flow to simulate varying levels of humidity using a standard chemical bubbler. The input for a single-sensor recognition system was in the form of 4 s exposures, while for the multi-sensory system sensors were exposed to analyte until they reached a steady-state.

**Figure 7.**
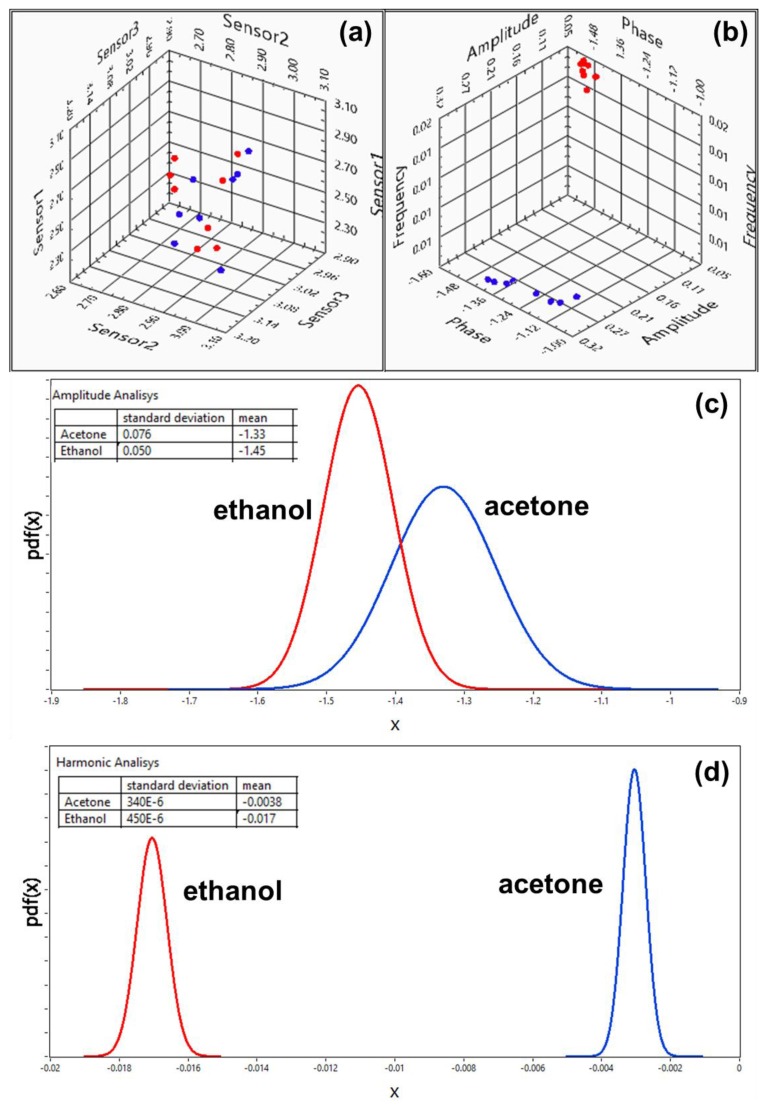
Separation of classes in the (**a**) hyperspace of steady-state responses of multi-sensory system (**b**) sub-space of the first harmonic extracted from the transient response. Probability density for acetone and ethanol for steady-state (**c**) and harmonic (**d**) analysis. Coordinate *x* was obtained by transformation of coordinates according to Fisher's criteria of LDA.

The extracted features for 16 exposures (eight exposures to acetone and eight to ethanol) for both configurations are shown in Figure 7a,b. For each analyte, the humidity level from exposure to exposure was adjusted by ∼3% in the range between 60% and 85%. Figure 7a shows the amplitude output of a multisensory system, and Figure 7b shows the sub-space of the first harmonic extracted from the transient response. Quantitatively, the harmonic and amplitude recognition techniques can be compared by using probability density functions (PDF). By decreasing the amount of dimensions and projecting the data points into C-1 dimensional space where C is the number of classes, we maximize the separation between classes and minimize the separation within the classes. Figure 7c,d show the probability density for acetone and ethanol as functions of coordinate *x* that was obtained by transformation of coordinates according to Fisher's criteria of LDA [3,17]. As it can be seen from the figure, the steady-state amplitude analysis leads to wide and overlapping distributions for two classes (Figure 7c), while the harmonic analysis generates well-separated narrow peaks (Figure 7d). The insets in the graphs show the mean values and standard deviations for each of the distributions. Also, in order to provide a quantitative evaluation of the steady-state and harmonic analysis, we compared a parameter:

(5) $$J=\frac{\text{sum of standard deviations of the classes}}{\text{Distance between the classes}}$$

which can be used to determine the discrimination power of the method [3]. The result of this comparison is shown in the Table 1. The parameter *J* is ∼ 17 times higher for harmonic analysis, than for steady-state amplitude analysis. Therefore, the variations in the level of humidity makes the separation of classes much less pronounced in the steady-state analysis, while through spectral analysis, the separation of classes remains much more stable.

## 5. Conclusions

The application of spectral analysis to the response signals of conductometric sensors was discussed. The analysis was demonstrated on the example of an ALD-fabricated ZnO chemiresistor, upon exposure to short 0.1 s pulses of analytes. The techniques of signal extraction, enhancement, spectral analysis and reconstruction were demonstrated. The recognition of analytes was accomplished by separating them in multi-dimentional hyperspace of extracted spectral characteristics using Quadratic Discriminant Analysis. The method helps to overcome common problems like short-term sensor drift and limited amount of the extracted signal features. The presented technique allows one to build a simple vapor recognition system based on a single broadly-tuned metal-oxide chemiresistor. The method can be further developed for the integrated sensor arrays for recognition of more complex analytes.

## Figures and Tables

**Table 1.** Quantitative comparison of discrimination capabilities of the steady-state and harmonic analysis at different humidity levels.

**Method**	**Distance between the Classes**	**Sum of Standard Deviations of Classes**	***J***
Harmonic Analysis	0.0132	790 × 10^−6^	16.709
Steady-state Amplitude Analysis	0.122	126 × 10^−3^	0.968
